# Short-term Effects of a Smoking Prevention Website in American Indian Youth

**DOI:** 10.2196/jmir.1682

**Published:** 2012-06-01

**Authors:** Deborah J Bowen, Patricia Nez Henderson, Jessica Harvill, Dedra Buchwald

**Affiliations:** ^1^School of Public HealthBoston UniversityBoston, MAUnited States; ^2^Black Hills Center for American Indian HealthRapid City, SDUnited States; ^3^State of Alaska Department of Health and Social ServicesSection of Chronic DiseaseAnchorage, AKUnited States; ^4^University of Washington School of MedicinePartnerships for Native HealthSeattle, WAUnited States

**Keywords:** Smoking prevention, Native American, eHealth, intervention, cultural appropriateness

## Abstract

**Background:**

The rate of smoking commercial tobacco products among American Indian youth is double the rate for white youth. Interventions are needed to reduce this disparity.

**Objective:**

To test the feasibility of a Web-based intervention to influence attitudes toward and intentions about smoking cigarettes among American Indian youth who attended a Native summer camp in the Northern Plains.

**Methods:**

The study website, the SmokingZine, was originally developed and tested in Canadian youth, then adapted to be appropriate for American Indian youth. We conducted a randomized controlled trial to test the influence of exposure to the adapted SmokingZine website on smoking attitudes and behaviors among American Indian youth 12–18 years of age. Participants assigned to the intervention group were given access to the website for 1 hour per day during their camp experience and asked to sign in to the site and use it. Control group participants were not given access to the site.

**Results:**

A total of 52% of intervention youth signed in to the website at least once. Among nonsmokers, intentions to try a cigarette in the intervention group declined from 16% to 0%, and increased from 8% to 25% in the control group (*P *< .05). Compared with the control group, youth in the intervention group were more likely to help others quit (21 percentage point change in intervention versus no change in control; *P *< .05) and had less positive attitudes about the drug effects of smoking (–0.19 change in intervention versus 0.67 in control; *P *< .05).

**Conclusion:**

These data indicate that SmokingZine needs more long-term, rigorous investigation as a way to keep American Indian youth from becoming regular smokers. Because the intervention group could use computers only 1 hour per day, increasing access might result in more visits and a greater effect of the website on smoking behaviors.

## Introduction

Smoking tobacco is responsible for more premature morbidity and mortality in the United States than any other behavioral risk factor [1]. Nationally, rates of smoking, as well as other forms of tobacco use, such as chewing tobacco, are substantially higher among American Indians than white people [2,3] and have not followed the downward trend observed over the past decade in the general US population [4,5]. Of particular concern, a recent report from the US Surgeon General on tobacco use among racial and ethnic minority people in the United States indicated that smoking rates among American Indian youth are double that of their white counterparts [4] and remain high even compared with their peers in other racial and ethnic minority groups [4].

High rates of smoking among American Indian youth may be due, in part, to the sovereign status of federally recognized tribes. Because tribal lands are often not subject to state tobacco control policies, American Indian youth have access to tobacco products at a very early age. Early initiation often leads to addiction and long-term abuse of tobacco [6,7], which in turn contribute to cardiovascular disease and lung cancer, the leading causes of death in the American Indian population [6,7]. Although these worrisome data highlight the need to develop or identify successful programs to both prevent and reduce smoking among American Indian youth, a Medline search conducted before initiating this project produced no adequately evaluated programs that targeted Native youth.

Increasing attention has focused on using the Web for smoking cessation support, including delivery of information, discussion groups, cognitive behavioral treatment, and self-help materials [8-14]. The SmokingZine website was originally developed explicitly for testing in urban Canadian youth [15]. Since 1995, the TeenNet project [14,16] at the University of Toronto has pioneered a combination of website development, community mobilization, and action research involving young people from diverse backgrounds in all stages of program design, development, and dissemination. In 2002, TeenNet began a randomized controlled trial of SmokingZine involving more than 1400 smoking and nonsmoking adolescents from participating Toronto-area high schools [8]. SmokingZine was both the largest randomized trial of an eHealth intervention for behavior change and one of the largest studies on adolescent smoking cessation [8,11]. In another clinical trial, the same intervention was efficacious in reducing smoking among middle-school youth in public schools [15].

Because of the success of the SmokingZine trial with youth from diverse backgrounds, adapting and testing this program for Native youth seemed promising. Because young people use the Web for health information [10], we conducted a randomized controlled study of this Web-based youth smoking prevention and cessation resource, SmokingZine [8,11], among American Indians 12–18 years of age attending summer camp in South Dakota. The outcomes of interest to our study were short-term smoking behaviors, attitudes about smoking, and usability of the Web tool [12]. Our findings are intended to identify issues for a full-scale efficacy study of the SmokingZine intervention in a larger sample.

## Methods

### Overview

We conducted a small randomized feasibility trial in one geographical site to test the 1-month effects of a Web-based intervention on smoking behaviors among American Indian youth 12–18 years of age. All participants completed a baseline survey and were then randomly assigned to one of two conditions: intervention (50%) or control. Intervention participants were asked to sign in to the website and use it, and were provided with regular access to the site during their camp experience. Control participants were not given access to the site. All participants completed a follow-up survey 1 month after randomization. We assessed and compared smoking behavior in both groups. In addition, we assessed attitudes and social behaviors that could be expected to change as a result of the intervention exposure. All control participants received access to the intervention after completion of the study. This study is reported in line with CONSORT requirements, and a flow chart and checklist were included in the review of the manuscript. We did not register this trial prospectively, as it was a feasibility trial, not a fully powered randomized trial.

### Setting and Participants

Each year more than 150 American Indian 6th to 12th graders from across South Dakota are selected to participate in a 6-week residential summer enrichment program in Rapid City, South Dakota. All nine Indian reservations located in South Dakota participate in the program, whose goal is to prepare students for success in college. During the 6 weeks, students attend classes in mathematics, science, English, computer use, and life skills. All camp attendees were eligible for this study, with a target sample size of 100 youth, based on a sample size calculation with power of 80% and a moderate effect size in intentions to smoke in the future.

### Recruitment and Consent Procedures

Working closely with the director of the summer program, we arranged to insert an informational sheet about the study into all correspondence mailed to the 164 selected students before the program started. During registration, our research staff set up an informational booth in the lobby of the students’ dormitory to describe the program to students and their parents. For students interested in participating, a consent form was read to both parents and students. A total of 113 students provided consent, completed the baseline assessment described below, and were randomly assigned to the intervention or control arm. Randomization was completed by assigning each consented and assessed youth to a study identification number that had been preassigned to either intervention or control status. Field staff were unaware of the preassignment.

### Intervention

Although it has a small cessation focus, the Web-based SmokingZine is primarily a smoking prevention intervention. It consists of a series of smoking educational modules that include culturally relevant contexts (a summer powwow) and images relevant to Native youth (eg, eagles, feathers, drums, and outdoor scenes). Our methods for developing and culturally adapting SmokingZine for use in this study have been previously published [16]. Briefly, we conducted talking circles with Native youth to ascertain their knowledge and attitudes about tobacco use, their opinions of SmokingZine, and their recommendations for changes. Talking circle participants provided useful information on the differences between ceremonial and commercial tobacco use, as well as their personal patterns and motivations regarding tobacco use. They also offered guidance on changes to the site that would make it more fun to use and more culturally appropriate. Examples of suggestions are replacing urban images with images of outdoor settings familiar to diverse Native youth, and replacing images of a shopping mall with images of a powwow. The research team made changes based on participants’ suggestions.

After randomization, control group participants were told that they would receive information about smoking at the end of the 6-week program. Intervention youth were given usernames and passwords to access the SmokingZine site and were instructed to use it as much as they wanted. Our intent was for the intervention participants to visit the site multiple times, with each visit lasting 10–20 minutes. The use of unique usernames and passwords enabled the research team to broadly track usage patterns of each participant over time. The research assistant maintained a list that linked each username to an identification number printed on the study questionnaire, enabling us to link site usage to baseline and follow-up data.

Each day of the 6-week program, students had 1 hour of computer time. Those in the intervention were encouraged to visit the SmokingZine site during this time. On the initial visit, the research assistant helped participants navigate to the site. Once participants signed in to the site, they were asked about their current smoking status. Then, based on their response, they were guided to educational modules about smoking prevention or cessation. The underlying message of these modules reinforced the values of nonsmokers and created a set of conditions to enhance smokers’ motivation and commitment to change. Toward the end of each session, modules helped smokers identify goals for behavior change and explore barriers to change, as well as providing advice and strategies where appropriate. During subsequent sessions, participants received additional information about smoking prevention or cessation.

### Assessments

All intervention and control youth were asked to complete a 30-minute paper-and-pencil survey on current smoking habits, intentions to smoke, and attitudes about smoking, both immediately after randomization and again 1 month later. We also approached youth who did not complete the entire survey to provide us with smoking outcomes only, to maximize response to a minimal outcome dataset.

The 87-item survey, called A Smoking Prevention Interactive Experience (ASPIRE) instrument, consisted of sociodemographic items, several scales, and individual items. Many of the items were derived from existing instruments, and others were created specifically for an interactive, multimedia smoking prevention and cessation curriculum for culturally diverse high-school students [9]. Attitudes were measured by a series of questions asking for the opinions of youth on various smoking-related issues. The primary outcome of this feasibility study was short-term smoking behaviors; secondary outcomes were attitudes about smoking and intentions to smoke.

At baseline, on the basis of questionnaire answers, students were categorized as current smokers (smoke every other week, smoke less than a pack a week, smoke a pack a week, smoke more than a pack a week, smoke a pack a day, or smoke more than a pack a day), former smokers (used to smoke regularly but quit), or nonsmokers, including never-smokers (never smoked even part of a cigarette) and experimenters (only smoked part of a cigarette or smoked only a few times).

### Analyses

We compared baseline demographic data between intervention and control participants, using *t *tests for continuous variables and chi-square tests for proportions to confirm that randomization was accomplished. We also examined website usage data for intervention participants to assess patterns and frequency of use.

Next, we examined changes in survey responses from baseline to 1-month follow-up in key smoking and related outcomes. We focused these analyses on selected intermediate outcomes of long-term smoking prevention, such as short-term smoking behavior changes, intentions to smoke in the future, and social actions that participants could take regarding smoking. We compared each of these variables from baseline to follow-up between intervention and control participants. Relatively few participants were missing at follow-up and, therefore, we used no imputation method.

We also performed a factor analysis on the attitude questions in the ASPIRE instrument to determine whether we could identify any groupings of questions for analysis. We identified three clusters, based on factor loadings of greater than 0.5, independent of loading on other factors. These were social issues (3 items, eg, “Kids who smoke have more friends”), drug effects (3 items, eg, “Smoking cigarettes relieves tension”), and negative effects) (9 items, eg, “Cigarette smoking is addictive”). We averaged the items in each cluster to form a score for each area. We then compared responses to attitudinal scores between intervention and control youth from baseline to follow-up.

## Results

The study team invited 164 young people to participate, and recruited and randomized 113 (68.9%). The mean age of participants in the intervention and control arms did not differ (mean of 14.8 vs 14.4 years). Of the 113 who completed the baseline measures, we were able to collect smoking follow-up data on 102 (90.3%). The intervention and control groups did not differ in the proportion who provided follow-up data (n = 49, 92% versus n = 44, 90%). Likewise, baseline demographic data and smoking behavior were similar between youth who completed and those who did not complete the follow-up assessment.

In terms of use, 10 (52%) intervention youth signed in to the website at least once. Of these, 39 (80%) used the site only once, and 5 (20%) used it 2 or more times. Overall, intervention users rated the site positively on the survey: 16 (33%) rated it as very useful and 27 (54%) rated it as somewhat useful. A large proportion of intervention users indicated that use of the site made them think very differently 22 (44%) or somewhat differently 19 (38%) about smoking. Finally, most of the intervention users indicated that use of the site would very much (36, 74%) or somewhat (3, 11%) keep them from smoking in the future.

Table 1 presents the characteristics of the 113 participants randomly assigned in the study. Baseline demographic characteristics did not differ across the intervention and control groups. Most participants reported their ethnic status as Native (95/113, 84%), with a preponderance of girls over boys (69, 61%), and a majority with at least one smoking parent (75, 66%).

**Table 1:** Characteristics of American Indian youth participating in a feasibility trial of the SmokingZine website.^a^

Characteristic		Intervention group (n = 64), n (%)	Control group (n = 49), n (%)	*P *value
Female		34 (53%)	35 (71%)	>.22
**Race**				>.86
	American Indian	58 (91%)	37 (76%)	
	White	2 (3%)	7 (14%)	
	Other	4 (6%)	5 (10%)	
**Age (years)**				>.35
	£13	7 (11%)	6 (21%)	
	14–26	57 (89%)	43 (88%)	
	≥27	0	0	
**Household size**				>.31
	£5	31 (48%)	28 (57%)	
	≥6	33 (51%)	21 (43%)	
**Mother smokes**				>.18
	Yes	28 (45%)	30 (61%)	
	Never smoked	16 (26%)	13 (27%)	
	Quit smoking	16 (26%)	6 (12%)	
	Don’t know	2 (3%)	0	
**Father smokes**				>.40
	Yes	25 (40%)	16 (33%)	
	Never smoked	16 (25%)	14 (29%)	
	Quit smoking	15 (24%)	10 (21%)	
	Don’t know	7 (11%)	7 (15%)	
**Parental marital status**				>.71
	Married	27 (42%)	22 (45%)	
	Separated	15 (23%)	12 (25%)	
	Divorced	12 (19%)	7 (14%)	
	Single	7 (11%)	5 (10%)	
	Widowed	1 (2%)	1 (2%)	
	One or both deceased	2 (3%)	2 (4%)	

^a ^Percentages are computed with 1–2 missing values excluded; sum of percentages may not total 100% due to rounding.

Table 2 presents data on smoking status and intent for the intervention and control groups at baseline and 1-month follow-up. Differences including smokers and former smokers were not tested statistically due to small sample sizes. Rates of current smoking at baseline among all youth were relatively low: almost 80% were never-smokers. The proportions of youth in the *never*, *current*, and *former *categories of smoking did not change from baseline to follow-up. The number of intervention youth who reported helping someone quit smoking increased over time. In contrast, the number in the control group did not change(6 versus 0, *P *< .05). Likewise, among nonsmokers, the number who intended to try a cigarette in the intervention group declined from 7to 0 and increased from 3 to 8 in the control group (*P *< .05). Finally, the other smoking-related variables did not differ across the intervention and control groups.

**Table 2:** Effects of exposure to the SmokingZine intervention on smoking-related variables.^a^

Question		Intervention group		Control group		*P *value
		Baseline (n = 64), n (%)	Follow-up (n = 59), n (%)	Baseline (n = 49), n (%)	Follow-up (n = 44), n (%)	
**Smoking status**						.58
	Nonsmoker	51 (85%)	47 (83%)	38 (83%)	35 (80%)	
	Current smoker	5 (8%)	3 (5%)	2 (4%)	2 (4%)	
	Former smoker	4 (6%)	7 (12%)	6 (13%)	6 (15%)	
	Missing data	4	2	3	2	
*All youth* **...Tried to help someone quit smoking**						<.01
	Yes	46 (70%)	52 (91%)	28 (58%)	28 (58%)	
	No	20 (30%)	5 (9%)	20 (42%)	15 (42%)	
*Former smokers* **...At any time during the next year, do you think you will smoke?**						.62
	Yes	1 (10%)	2 (29%)	6 (100%)	6 (100%)	
	No	9 (90%)	5 (71%)	0	0	
*Nonsmokers* **...In the next year, do you think you will try smoking a cigarette?**						<.02
	Yes	7 (16%)	0	3 (8%)	8 (25%)	
	No	37 (84%)	38 (100%)	34 (92%)	27 (75%)	
*Never-smokers* **...If one of your best friends offered you a cigarette, would you smoke it?**						.26
	Yes	1 (2%)	2 (5%)	1 (3%)	0	
	No	43 (98%)	42 (96%)	36 (97%)	32 (100%)	

^a ^Percentages were computed with 1–3 missing values excluded; sum may not total 100% due to rounding.

Table 3 presents data on the attitudinal changes from baseline to follow-up in both the intervention and control groups. Scores for the drug effects and negative effects clusters (described above), but not for social issues, changed significantly from baseline to follow-up in the intervention group but not in the control group, in a direction consistent with the intended effects. Intervention youth felt less positively than the control youth about the drug effects and more negatively about the adverse effects of tobacco.

**Table 3:** Key short-term attitudes (range 1–4) related to smoking for the intervention and control groups.^a^

Attitude cluster		Intervention group (n = 64)		Comparison group (n = 44)		*P *value
		Mean	SD	Mean	SD	
**Social issues**						.23
	Baseline	1.32	0.18	1.20	0.26	
	Follow-up	1.11	0.27	1.31	0.28	
**Drug effects**						<.04
	Baseline	1.86	1.1	1.67	1.1	
	Follow-up	1.1	1.2	1.77	1.2	
**Negative effects**						<.02
	Baseline	4.20	1.7	4.05	1.9	
	Follow-up	4.8	1.4	4.01	1.8	

^a ^Means are computed with all follow-up missing values excluded; n may not total 100% due to rounding or missing variables.

## Discussion

The purpose of the present study was to test several aspects of the feasibility of the adapted Web-based SmokingZine intervention for American Indian youth. The intervention was feasible and acceptable for at least half of the participants, and at least somewhat effective for American Indians 12–18 years of age. The trial was successfully implemented with little oversight or structure, among staff and youth generally unfamiliar with research projects or randomized designs. Moreover, most students (69%) in the summer camp participated in the study, indicating that a larger randomized test of this, or a similar, Web-based intervention is logistically possible. This evidence of feasibility is critical, because concerns about the adequacy of prior study designs have plagued the literature on youth smoking prevention [13]. We also were pleased that a substantial proportion of the intervention group found the website interesting and useful.

Only 52% used the site, although a much smaller proportion used it on more than one occasion. This is a common occurrence among Web-based studies of youth, where actual use of the website in randomized trials is often low [17]. In this study the investigators identified some engagement strategies for youth to increase their use of and engagement in the Web-based activities, and this is a promising avenue for future research and implementation. Future studies should consider strategies to increase the proportion of randomly assigned participants who visit the SmokingZine website more than once, for instance by structuring or scheduling use, providing incentives for use, or increasing access to computers. Qualitative work has identified the multiple ways in which youth engage in using eHealth technologies to gain information [18], and some of these ways are relevant to the present application. For example, finding personalized and tailored information was a need expressed by youth in the focus groups, and the SmokingZine website could be more tailored to individual users’ needs and data and marketed as such.

In regard to marketing, in our study, the moderate amount of website use might have resulted from the strict limitation on computer access, suggesting that liberalizing the amount of computer time might enhance the effect of the intervention. If the program were implemented in schools, there would be supervised and required time for sessions on the computer that would likely promote the visiting of the website by youth during school hours.

Given our experiences with this study, we propose that a group design would be ideal, given the opportunity for sharing site passwords as well as information and materials pertinent to the intervention among youth in the same physical location. The popularity among youth of social networking sites such as Facebook also suggests that a group design would be appropriate for a larger trial. As well, in a study with a longer follow-up period, preexisting friendships and social networks would result in youth using the site together. We did not observe joint use in our study, but in settings where computers are publicly available, such as schools and community centers, joint use could occur. The use of group randomized trials, in which the unit of intervention is the group, would help to minimize this problem. Finally, biochemical validation could be used to verify smoking outcomes in any future rigorous efficacy trials aimed at American Indian youth.

In the short run, the intervention did not directly affect smoking behavior, although it did alter intentions to use tobacco among never-smokers. This finding is encouraging for future efficacy trials, as the relatively high rates of smoking in later adolescence occur in both early- and later-starting smokers. This type of intervention may thus be best suited for young people who are still irregular smokers or nonsmokers at the time of exposure. The intervention did change social interactions involving smoking, such as offering to help others quit. This may be the most intriguing and important finding, as tobacco use is a socially driven phenomenon [19] that could be prevented by breaking down socially normative perspectives on tobacco.

Given our small sample size, the lack of effects on smokers may simply indicate lack of power coupled with low baseline rates of smoking. These low baseline rates, in turn, likely reflect the biases related to being selected to attend a camp for American Indian youth. For example, the students had to have a high grade-point average and were highly recommended by their school counselor and teachers. Smoking is more frequent among Native youth with lower academic performance, who perhaps were not widely represented at this camp [16]. In other community settings, we would be more likely to encounter a wider variety of Native youth, including more regular smokers. This is a major limitation to the generalizability of the present study data, in that less achievement-oriented Native youth might not respond to this website. The structured nature of the summer camp is also a limitation of the present study, as youth in the camp are not near their regular and ongoing social influences, and so the artificial structure of the camp interactions might alter the effects of the intervention.

Besides the highly selected sample of American Indian youth attending summer camp, other limitations of this feasibility study include the short duration of the follow-up and the lack of qualitative data to enhance our understanding of quantitative survey findings. As well, a key limitation was the camp’s rigorous control of access to the Web, allowing youth only 1 hour of computer use each day. We expect that participants neither visited the site as much nor stayed as long as they might have with longer or unlimited Web access, thus diluting the potential impact of the intervention. There was always the potential in this individually randomized design for contamination to occur between intervention and comparison youth, discussing smoking and even using the website together. Finally, despite our best efforts, the randomization intended to be evenly divided between intervention and comparison youth was not, and so more rigorous randomization procedures will need to be instituted for the next study. Even so, strengths include the use of a previously tested intervention, a documented adaptation process, and a rigorous feasibility design.

Future efforts should pay closer attention to cessation among subsets of youth smokers, because American Indians who smoke in youth are most likely to become regular adult smokers [20]. If cessation needs more emphasis, the intervention website will need to be enhanced.

These findings partially support the potential of the SmokingZine tool for future research. We believe this feasibility study has set the stage for future intervention research into tobacco use reduction among American Indian and Alaska Native youth. Online tobacco control efforts have started to emerge [21-24], but none to date have been designed for or targeted at American Indians and Alaska Natives. In a similar vein, some authors have called for more use of eHealth technology to enhance the health of Native people, both inside and outside the Indian Health Service [19].

## Abbreviations

ASPIRE: A Smoking Prevention Interactive Experience
